# Screening for functional IRESes using α-complementation system of β-galactosidase in *Pichia pastoris*

**DOI:** 10.1186/s13068-019-1640-3

**Published:** 2019-12-27

**Authors:** Yide Huang, Yafei Zhang, Suhuan Li, Ting Lin, Jingwen Wu, Yao Lin

**Affiliations:** 1 Provincial University Key Laboratory of Cellular Stress Response and Metabolic Regulation, College of Life Sciences, Fujian Normal University, Fuzhou, 350007 China; 2 Key Laboratory of Optoelectronic Science and Technology for Medicine of Ministry of Education, Fujian Normal University, Fuzhou, 350007 China

**Keywords:** *Pichia pastoris*, IRES, β-Galactosidase, α-Complementation, Multigene co-expression

## Abstract

**Background:**

*Pichia pastoris* is becoming a promising chassis cell for metabolic engineering and synthetic biology after its whole genome and transcriptome sequenced. However, the current systems for multigene co-expression in *P. pastoris* are not efficient. The internal ribosome entry site (IRES) has an ability to recruit the ribosome to initiate protein synthesis by cap-independent translation manner. This study seeks to screen IRES sequences that are functional in *P. pastoris*, which will allow *P. pastoris* to express multiple proteins in a single mRNA and increase its efficacy as a platform for metabolic engineering and synthetic biology.

**Results:**

In order to efficiently screen the IRES sequences, we first set out to create a screening system using *LacZ* gene. Due to the cryptic transcription of the *LacZ* gene, we established the α-complementation system of β-galactosidase in *P. pastoris* with the optimum length of the α-complementing peptide at ~ 92 amino acids. The optimal α-complementing peptide was then used as the second reporter to screen IRESes in the engineered GS115 expressing the corresponding ω-peptide. A total of 34 reported IRESes were screened. After ruling out all false positive or negative IRESes, only seven IRESes were functional in *P. pastoris*, which were from TEV, PVY, RhPV, TRV, KSHV, crTMV viruses and the 5′-UTR of the *YAP1* gene of *S. cerevisiae*.

**Conclusions:**

We showed here that α-complementation also works in *P. pastoris* and it can be used in a variety of in vivo studies. The functional IRESes screened in this study can be used to introduce multiple genes into *P. pastoris* via a prokaryotic-like polycistronic manner, which provided new efficient tools for metabolic engineering and synthetic biology researches in *P. pastoris*.

## Background

*Pichia pastoris* (today re-classified into the genus *Komagataella*), a methylotrophic yeast, has become an important industrial microorganism for the production of heterologous proteins due to its rapid growth, high-density fermentation, simple genetic manipulation and capacity of posttranslational modification [1]. The simple genetic organization (such as haploid and lack of introns on most genes), no overflow metabolism and the methanol assimilation pathway of *P. pastoris* determine its potential as a chassis strain for metabolic engineering and synthetic biology which has already attracted a lot of research interest soon after the whole genome and transcriptome sequencing of *P. pastoris* [2–7].

In metabolic engineering and synthetic biology, the production of synthetic molecules often involves integration of multiple genes or even entire metabolic pathways into the host. For example, in order to realize secretion of a human glycoprotein from *P. pastoris*, a total of 14 genes on the human glycosylation pathways were integrated into the yeast genome [8]. In the production of riboflavin, a total of six key genes in the riboflavin biosynthesis pathway were integrated into *P. pastoris* genome, which effectively increased the yield of riboflavin [9]. However, the expression of entire heterologous pathways is technically challenging in *P. pastoris*. Therefore, how to better solve co-expression of multiple genes is important for *P. pastoris* to become a highly efficient platform for the application of metabolic engineering and synthetic biology.

The current common approach to express multiple genes in *P. pastoris* is to construct multiple expression vectors (each vector with one target gene) or multiple expression cassettes in an expression vector (each cassette consisting of a promoter, a target gene and a terminator) [10–12]. The method for expressing multiple genes by multiple vectors requires some vectors and selectable markers together with repeated rounds of transformation and screening during genetic manipulation, which greatly increases the difficulty of successful genetic engineering. Using multiple expression cassettes in one expression vector to express multiple genes increases the chance of homologous recombination due to the use of too many identical promoter and terminator sequences and often leads to genetic instability [13].

Some viruses infecting eukaryotic cells can use the unique mRNA sequences on their untranslated regions (UTRs) called internal ribosome entry sites (IRESes) to directly recruit ribosomes to start cap-independent internal initiation for their protein synthesis [14]. The IRES elements were firstly observed in polio virus and encephalomyocarditis virus mRNAs [15, 16]. The cap-independent internal initiation translation was found not only in virus mRNAs but also in the UTRs of some eukaryotic mRNAs [17, 18]. The properties of IRES elements recruiting translational machinery to initiate protein synthesis by passing the requirement for the 7-methyl-guanosine cap at the 5′-end of the mRNA and its associated protein factors allow the construction of a bicistronic or polycistronic expression system to express multiple protein products using a single promoter and terminator in a single plasmid [19–21]. It has been reported that the drawbacks of double transformation and selection can be avoided by using an IRES to construct bicistronic vector [18]. Therefore, IRES may provide an effective approach for multiple gene expression in *P. pastoris*.

Some studies demonstrated that the yeast *Saccharomyces cerevisiae* has a potential to use IRES elements to initiate the translation [22–25], suggesting that IRES elements also are active in lower eukaryotes like yeast. Moreover, the 5′-UTR of GPR1 mRNA from *S. cerevisiae* was demonstrated to possess IRES activity [18]. After sequencing the *P. pastoris* transcriptome, Liang S et al. analyzed the 5′-UTR of 914 genes and first identified that the 5′-UTRs of GCN2 and KOG1 may contain functional IRESes [4]. These studies suggested that *P. pastoris* also has the potential to initiate the translation by cap-independent internal initiation. Many IRESes from viral RNA and cellular 5′-UTR functioning in *S. cerevisiae*, insects, plants or animals have been identified since the first IRES was discovered in 1988. They were well summarized in the review by Baird et al. [14]. It is still unknown whether the cellular protein synthesis apparatus in *P. pastoris* can efficiently handle these IRESes from other species, especially viruses. In this paper, we used a bicistronic reporter system with EGFP as the first reporter and α-peptide of β-galactosidase as the second reporter to screen 29 viral IRESes and five cellular IRESes from *S. cerevisiae* to find out functional IRESes in *P. pastoris*.

## Results

### Cryptic transcription of the *LacZ* gene in *P. pastoris*

**Fig. 1:**
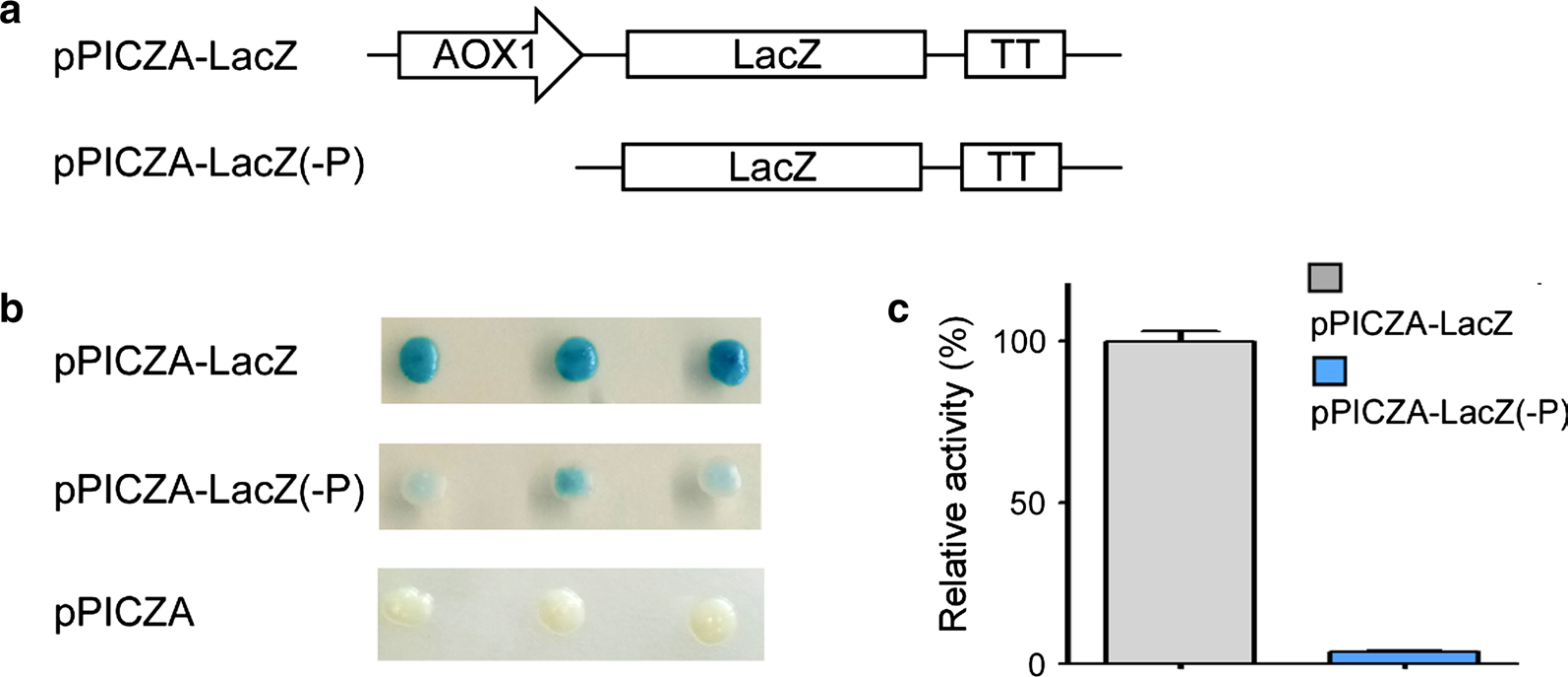
Background activity of *LacZ* gene in *P. pastoris*. **a** Schematic diagram of expression vectors with or without AOX1 promoter. **b** Analysis of enzymatic activity of β-galactosidase on BMMY plates containing X-Gal. The vector pPICZA-LacZ that the transcription of *LacZ* gene is driven by AOX1 promoter was used as positive control, and empty vector pPICZA was used as negative control. **c** Quantitative analysis of enzymatic activity of β-galactosidase

*Escherichia coli* β-galactosidase [EC 3.2.1.23] is capable of decomposing X-gal (a colorless soluble compound) to form a galactose and a substituted indole that spontaneously dimerizes to give an insoluble, blue product. The microorganism expressing β-galactosidase forms blue colonies on the solid medium bearing X-gal, which makes β-galactosidase a convenient and effective reporter in microbial research [26]. Based on this property of β-galactosidase, we planned to use β-galactosidase as a reporter in bicistronic system to indicate the function of IRESes in *P. pastoris*. Several researchers have reported that cryptic promoters in plasmid backbones or reporter genes are able to drive transcription of reporter genes to generate unwanted aberrant transcripts [27–29]. If the reporter gene in reporter constructs can be transcribed by cryptic promoter in plasmid backbone or reporter gene itself, it will reduce the reliability of the experimental results especially in screening for functional IRESes. Therefore, we first verified whether the *LacZ* gene can be transcribed by possible cryptic promoter in plasmid backbone or reporter *LacZ* gene in *P. pastoris*. The promoter driving the expression of *LacZ* gene in the vector was removed to construct a promoterless vector pPICZA-Lac(-P) (Fig. 1a). The pPICZA-Lac(-P), positive vector pPICZA-LacZ and negative vector pPICZA were transformed into *P. pastoris* GS115, respectively. We found that the background activities of β-galactosidase were detected in GS115 with the transformation of pPICZA-Lac(-P) (Fig. 1b, c), indicating that there may be a cryptic promoter in plasmid backbone or *LacZ* gene to drive *LacZ* gene expression in *P. pastoris*.

### α-complementation of β-galactosidase in *P. pastoris*

**Fig. 2:**
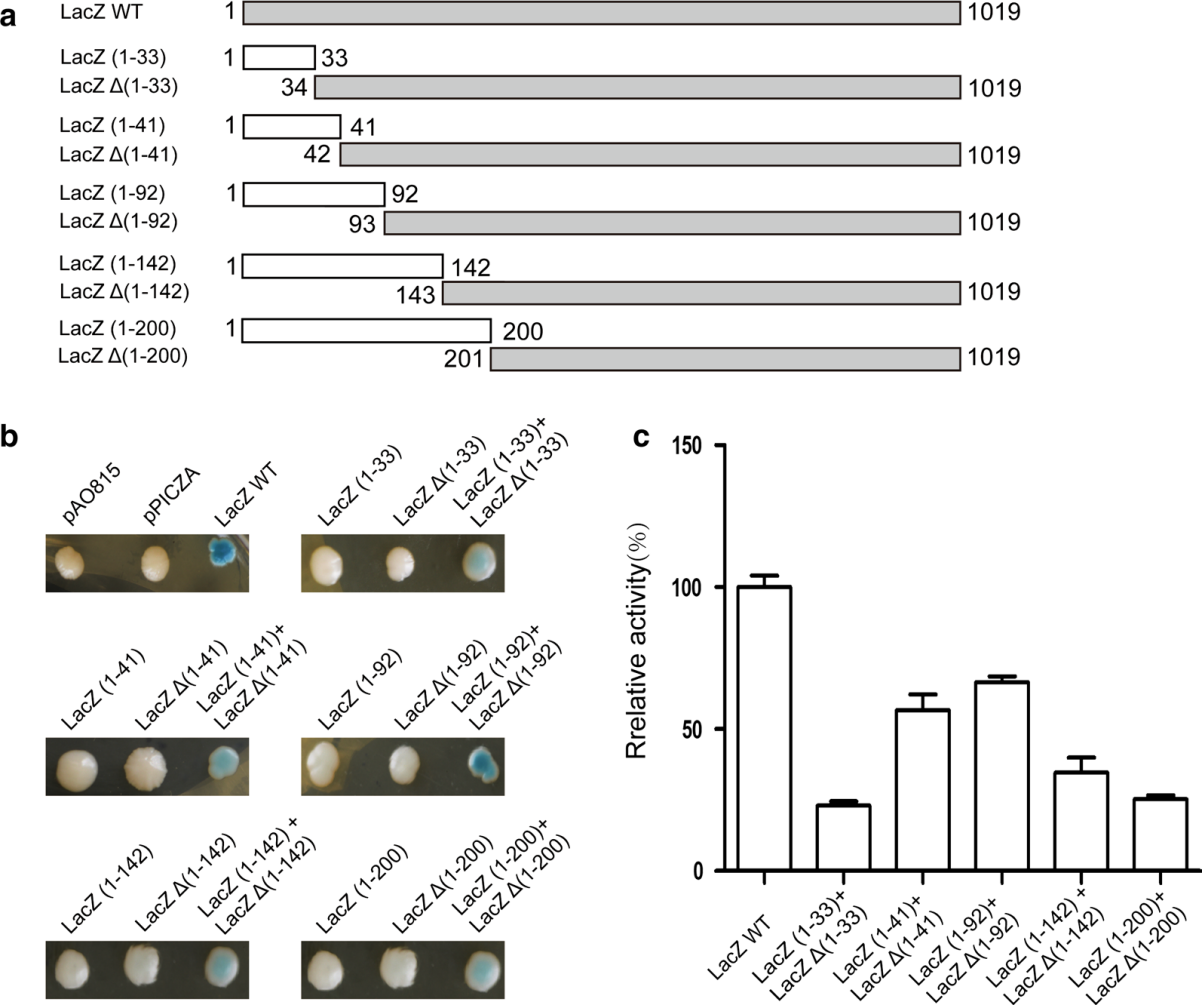
Development of the α-complementation of β-galactosidase in *P. pastoris*. **a** Schematic diagram of vectors containing α-peptides and ω-peptides with different lengths. **b** Detection of β-galactosidase activity on plates containing X-gal. Empty vector pAO815 and pPICZA as negative controls; full-length *LacZ* gene (LacZ WT) as a positive control. **c** The relative activities of β-galactosidase in the α-complementation systems with different lengths of α-peptide and ω-peptide. Normalization was performed with full-length β-galactosidase (LacZ WT) activity at 100%

Based on our results, screening for a functional IRESes directly using the full-length *LacZ* gene is not practical due to the cryptic transcription of *LacZ* gene in *P. pastoris*. As is known, the β-galactosidase is required to form a homotetramer to acquire enzymatic activity, and the formation of the homotetramer depends on the amino acid sequence of so-called α-peptide or α-donor at the N-terminus of β-galactosidase. If α-peptide is removed from the full-length enzyme, the remaining amino acid sequence (so-called ω-peptide or α-acceptor) does not form tetramers and has no enzymatic activity [30]. In an in vitro experiment, the activity of β-galactosidase could be restored in the presence of both the N-terminal α-peptide and the C-terminal ω-peptide, a phenomenon known as α-complementation, which has been used in bacteria and mammals [31–33]. If the α-complementation of β-galactosidase can be established in *P. pastoris*, it may solve the problem of cryptic transcription of *LacZ* gene through a bicistronic vector system. In order to establish an efficient α-complementation system, the expression vectors with α-peptide and ω-peptide of different lengths were constructed (Fig. 2a), and the corresponding vectors containing encoding sequences of α-peptide and ω-peptide were co-transformed into *P. pastoris*. The α-peptide with a length of 1–92 amino acids had the best complementary effect, and its enzymatic activity could reach about 65% of the full-length β-galactosidase activity (Fig. 2b, c). The size of the bicistronic vectors was particularly important in this study because 34 IRESes would be cloned into the bicistronic vectors and transformed into *P. pastoris*, the ligation and cloning efficiencies of which usually drop with the increase in vector size. Therefore, we decided to integrate the DNA sequence encoding ω-peptide which has the larger molecular weight into the *P. pastoris’s* genome to construct a transgenic strain expressing ω-peptide and clone the DNA sequence encoding the α-peptide as the second reporter gene inserted behind the IRESes sequence in the bicistronic vector. The encoding sequence of the α-peptide bearing 1–92 amino acids was then used as a reporter gene in the bicistronic vector for functional IRESes screening in the following studies.

### Screening for functional IRESes in *P. pastoris*

**Fig. 3:**
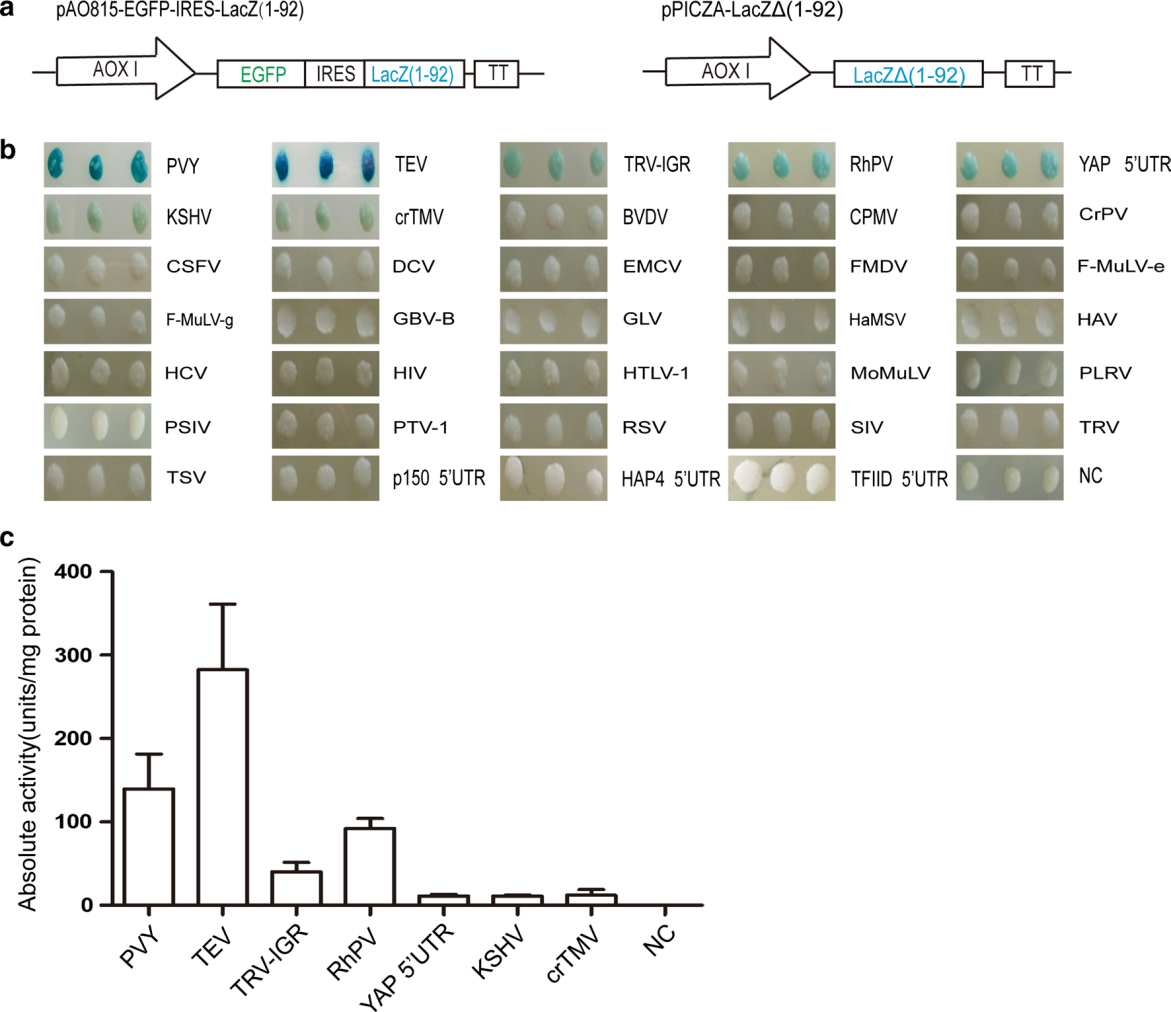
Screening for functional IRESes in *P. pastoris*. **a** Schematic diagram of the plasmid vectors. **b** Screening on the X-gal plate for functional IRESes in *P. pastoris*. Three different colonies were shown for each IRES. **c** Quantitative detection of β-galactosidase activity for seven functional IRESes in *P. pastoris*. The strains transformed with empty vector were used as negative control (NC)

To screen functional IRESes in *P. pastoris*, 34 IRES sequences that are functional in plants, mammals or *S. cerevisiae* were selected, 30 of which came from viruses and four from the 5′-UTR region of *S. cerevisiae’s* genes (Tables 1, 2 and Additional file 1). The 34 IRES sequences were cloned into the bicistronic vectors between the first reporter (EGFP) and the second reporter (α-peptide (1–92aa) of β-galactosidase). The encoding sequence of β-galactosidase ω-peptide was cloned into another vector for the construction of a transgenic strain GS115-LacZΔ (1–92) expressing ω-peptide (Fig. 3a). The 34 bicistronic vectors were separately transformed into GS115-LacZΔ (1–92), and the function of every IRES in *P. pastoris* was examined by detecting β-galactosidase activity. The β-galactosidase activities were only detected in 7 out of the 34 transformed strains, the IRES sequences in which came from TEV, PVY, RhPV, TRV, KSHV, crTMV viruses and the 5′-UTR of the *YAP1* gene of *S. cerevisiae* (Fig. 3b, c). Of note, the transgene expression was often affected by integration location, transgene silencing, transgene loss, etc. [34–36]. Although the β-galactosidase activities were not detected in the other 27 transformed strains, it is possible that it was caused by transgene silencing or loss, rather than lack of IRES activity. To eliminate the false negative possibilities, the protein expression of the first reporter EGFP was examined by western blot. The EGFP proteins were all expressed in the 27 IRESes, which further confirmed that these 27 IRESes had no functional activity in *P. pastoris* (Additional file 2: Figure S1A). Among the seven functional IRESes, their β-galactosidase activities were different. Studies have shown that copy number of transgenes affected their expression in *P. pastoris* [37, 38]. To make sure that the difference of IRES activities was not caused by copy number difference of transgenes, relative copy numbers of the α-peptide sequence were examined by genomic real-time PCR [39]. There was no significant difference in the copy numbers of the seven IRESes (Additional file 2: Figure S1B), indicating that the differences of β-galactosidase activity are caused by translation efficiencies driven by different IRES sequences in *P. pastoris*.

**Table 1:** Viral IRES information involved in this study

Virus abbreviation	Full name of the virus	Family	Viral host	GI	Position of IRES in viral genomic sequence
BVDV	Bovine viral diarrhea virus	*Flaviviridae*	Cow	9,836,967	1–385
CPMV	Cowpea mosaic virus	*Comoviridae*	Plant	58,910	161–514
CrPV	Cricket paralysis virus	*Dicistroviridae*	Insect	8,895,506	6025–6216
crTMV	Tobacco mosaic virus	*Tobamovirus*	Plant	488,713	5456–5606
CSFV	Classical swine fever virus	*Flaviviridae*	Pig	12,584,212	1–376
DCV	Drosophila C virus IRES2	*Dicistroviridae*	Insect	2,388,672	6080–6266
EMCV	Encephalomyocarditis virus	*Picornaviridae*	Human	9,626,692	260–836
FMDV	Foot and mouth disease virus	*Picornaviridae*	Mammals	61,076	252–716
F-MuLV-g	Friend murine leukemia virus	*Retroviridae*	Mouse	61,544	1–357
F-MuLV-e	Friend murine leukemia virus	*Retroviridae*	Mouse	61,544	5492–5780
GBV-B	Hepatitis virus insolated B	*Flaviviridae*	Primates	13,162,187	23–448
GLV	Giardia lamblia virus	*Totiviridae*	Giardia	1,352,866	1–369
HaMSV	Harvey murine sarcoma virus	*Retroviridae*	Rat	207,672	25–543
HAV	Hepatitis A virus	*Picornaviridae*	Human	329,585	150–720
HCV	Hepatitis C virus	*Flaviviridae*	Human	12,831,192	1–344
HIV1	Human immunodeficiency virus 1 GAG	*Retroviridae*	Human	4,558,520	335–808
HTLV-1	Human T cell Lymphotropic virus 1	*Retroviridae*	Human	221,866	354–621
KSHV	Kaposi-sarcoma-associated herpesvirus	*Herpesviridae*	Human	2,065,526	123,206–122,709
MoMuLV	Moloney murine leukemia virus	*Retroviridae*	Mouse	331,973	912–1040
PLRV	Potato leafroll virus	*Luteoviridae*	Plant	222,301	1513–1728
PSIV	Plautia stali intestine virus	*Dicistroviridae*	Insect	2,344,756	5949–6195
PTV-1	Porcine teschovirus serotype 1 talfan	*Picornaviridae*	Pig	13,111,645	146–434
PVY	Potato virus Y	*Potyviridae*	Plant	61,450	1–187
RhPV	*Rhopalosiphum padi* ORF2	*Dicistroviridae*	Insect	2,911,298	6327–7112
RSV	Rous sarcoma virus	*Retroviridae*	Chicken	2,801,459	230–382
SIV	Simian immunodeficiency virus	*Retroviridae*	Primate	334,657	507–1043
TEV	Tobacco etch virus	*Potyviridae*	Plant	335,201	2–147
TRV	Triatoma virus	*Dicistroviridae*	Insect	6,003,484	1–551
TRV-IGR	Triatoma virus	*Dicistroviridae*	Insect	6,003,484	5934–6111
TSV	Taura syndrome virus	*Dicistroviridae*	Penaeus vannamei	14,701,764	6741–6952

**Table 2:** Gene 5′-UTR information involved in this study

Gene name	Function	Organism	GI	Position of IRES in mRNA sequence
YAP1	Yes-associated protein 1 transcriptional activator	*S. cerevisiae*	4797	1–373
p150	Translation initiation factor homolog eIF4G	*S. cerevisiae*	1,323,279	14,111–14,641
HAP4	Transcription factor HAP4	*S. cerevisiae*	3762	1–503
TFIID	Transcriptional activator	*S. cerevisiae*	172,898	1–275

### Translational activity of the IRESes is not influenced by read-through, cryptic transcription or splicing

**Fig. 4:**
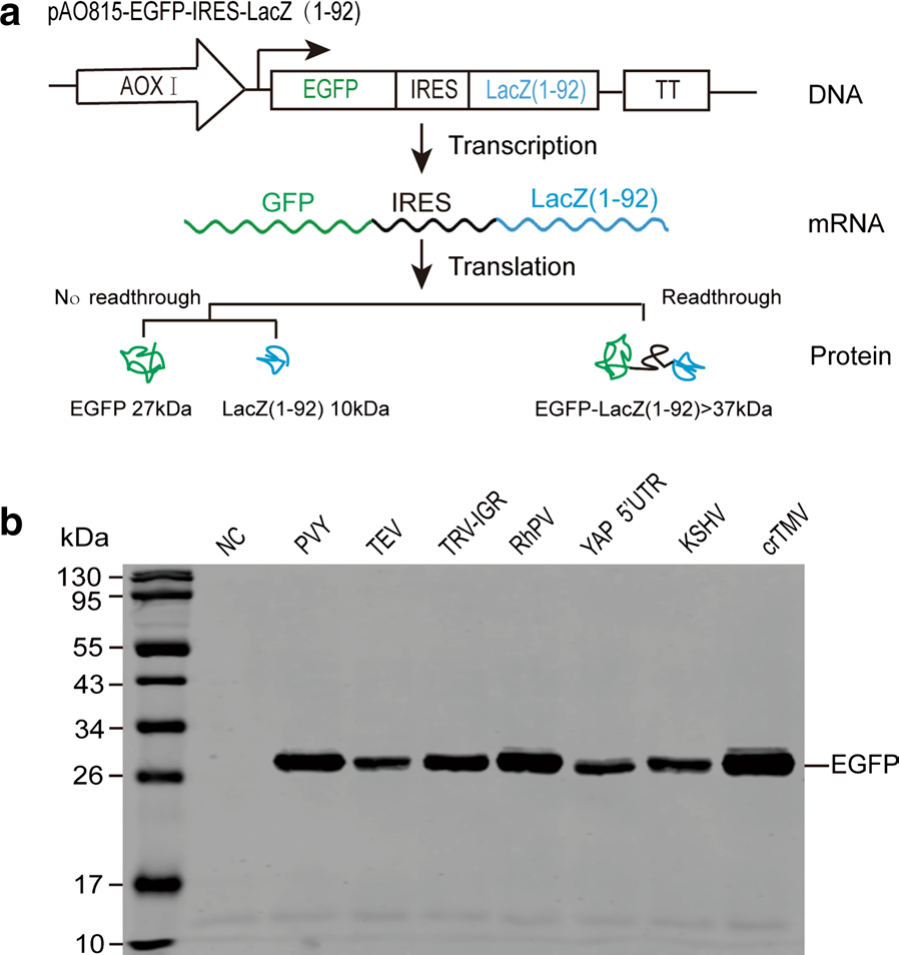
Exclusion of read-through of bicistronic mRNA. **a** Schematic diagram of proteins produced by non-read-through and read-through of bicistronic mRNA. **b** The fusion protein produced by read-through of bicistronic mRNA was excluded by western blot

To further confirm the activity of the seven IRES sequences, several potential false positive possibilities need to be ruled out. If the ribosomes were not stopped at the stop codon of the first reporter and were read through the bicistronic mRNAs to the second reporter during translation, fusion proteins of EGFP and β-galactosidase α-peptide (1–92aa) would be produced (Fig. 4a). The fusion proteins could also function in α-complementation of β-galactosidase, resulting in a false positive result. If fusion proteins of EGFP and β-galactosidase α-peptide (1–92aa) were produced, they would be larger than EGFP (26.9 KD) and detected by western blotting. The anti-EGFP antibody was used to examine the fusion proteins of seven transgenic strains with β-galactosidase activity. No fusion proteins were detected in all seven cases (Fig. 4b).

**Fig. 5:**
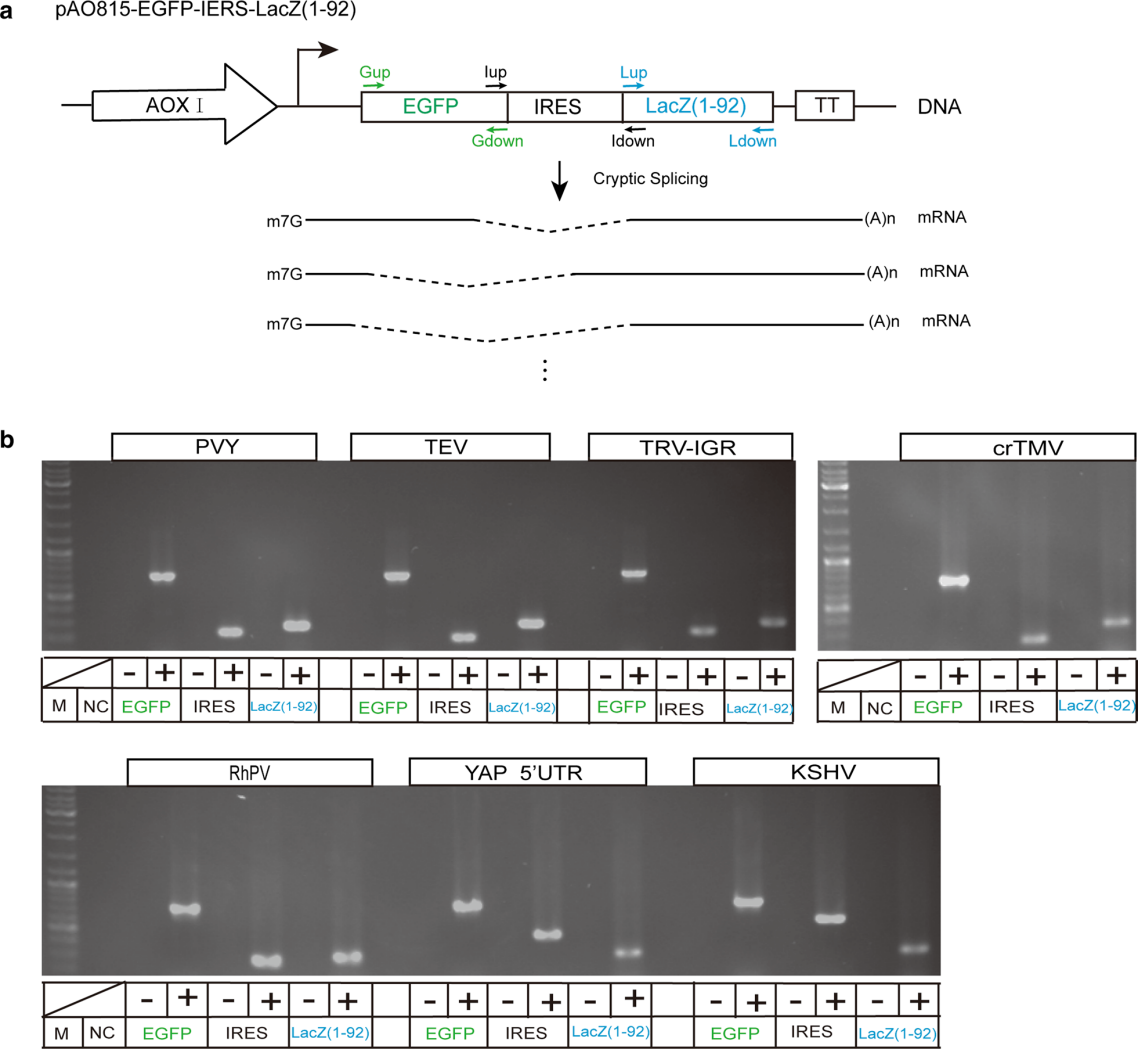
Detection of cryptic splicing in bicistronic mRNA by RT-PCR. **a** Schematic diagram of cryptic splicing and primer design. **b** Detection of RT-PCR products on agarose gel. NC, PCR negative control reaction without template. ∓, reactions without or with reverse transcriptase, respectively

The analysis of the genome sequences of *P. pastoris* showed that there are 5313 coding genes, of which 633 genes contained introns, indicating that *P. pastoris* has the ability for intron processing [40]. If there was a cryptic splicing site between the EGFP and LacZ (1–92) sequences in the bicistronic mRNA, the mRNA could be spliced by the splicing apparatus of *P. pastoris* to produce a monocistronic mRNA in which the IRES sequence was removed. In the monocistronic mRNA, the translation of the β-galactosidase α-peptide (1–92) would not be driven by the IRES in cap-independent translation but by cap-dependent translation (Fig. 5a). To exclude the possible occurrence of cryptic splicing in the bicistronic mRNAs, three pairs of overlapping primers (Gup and Gdown; Iup and Idown; Lup and Ldown) on the EGFP, IRES and LacZ (1–92) sequences were designed and used to detect mRNAs by RT-PCR. All three PCR products were correctly amplified, and the size of the products was the same as the expected size in all seven cases, indicating that there is no evidence for cryptic splicing or any kind of mRNA shortening in the bicistronic mRNAs (Fig. 5b).

**Fig. 6:**
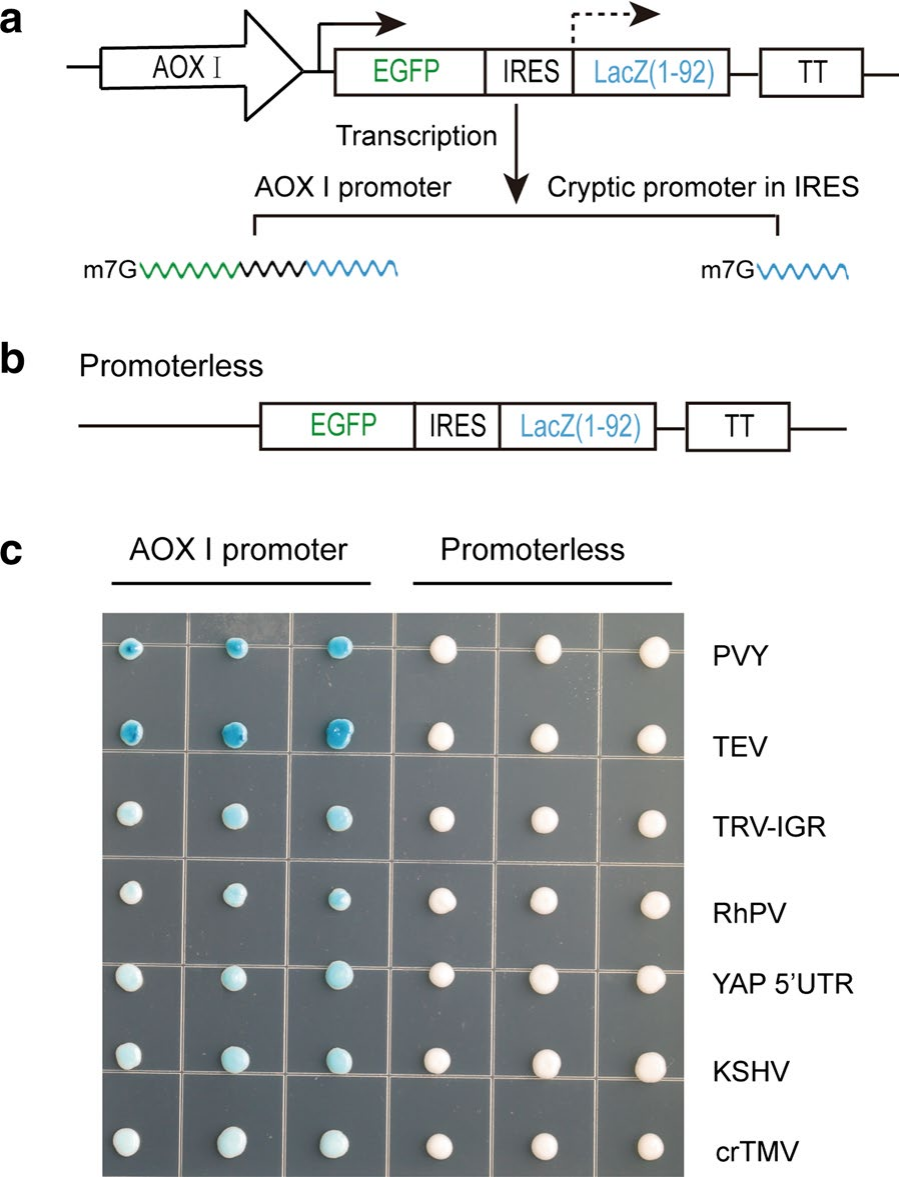
Detection of cryptic promoter using promoterless vector. **a** Schematic diagram of producing LacZ (1–92) transcript with 5′ cap by a cryptic promoter harbor in IRES sequence. **b** Schematic diagram of promoterless vector removed the AOX1 promoter. **c** Detection of expression of the second reporter LacZ (1–92) with or without AOX1 promoter

It is also possible that the expression of the reporter located in the second position is driven by cryptic promoter harbored in IRES sequence, but not the real activity of IRES [41, 42]. The internal initiation activities of IRESes observed in this study may be due to the cryptic promoters (Fig. 6a). To test whether a cryptic promoter was present in the IRES, the promoterless vectors without the AOX1 promoter were used (Fig. 6b). All seven IRESes (TEV, PVY, TRV, RhPV, KSHV crTMV and 5′-UTR of YAP1) could not induce the expression of LacZ (1–92aa) gene in the promoterless constructs (Fig. 6c), eliminating the possibility of a cryptic promoter within IRESes.

## Discussion

β-Galactosidase coding by *LacZ* gene from *E. coli* is very popular nowadays as a useful reporter in the research of microorganism due to its ability to hydrolyze the substrate X-gal to form blue colonies which is appropriate for screening. In this study, more than 30 IRES sequences coming from viruses and 5′-UTR of genes were examined in *P. pastoris*. Several published researches have reported that cryptic promoters or cryptic splicing sites present in plasmid backbone or reporter genes could generate unwanted aberrant transcripts which produce artifactual results including tests for IRES function [18, 27, 28]. In this study, we used promoterless vector to test possible aberrant transcript of *LacZ* gene in *P. pastoris* before IRES function was analyzed. The results clearly demonstrated that the strains transformed with promoterless vectors with *LacZ* gene possess β-galactosidase activity, indicating there probably could be cryptic promoter activity within the backbone of vector and/or reporter *LacZ*. It was reported that firefly luciferase gene from the common North American firefly *photinus pyralis*, another widely used reporter gene, also possesses a cryptic promoter activity that is detectable both in the budding yeast *S. cerevisiae* and in mammalian cells [27]. According to these findings, researchers must be aware of the unexpected transcription from cryptic or unusual sites bearing in the backbone of plasmid or reporter genes when the reporter system is employed to analyze cis-regulatory elements for the purposes of transcription or translation. Experiments carried out in microorganism are prone to be affected by cryptic transcription because they use shorter and simpler elements to initiate transcription compared to higher eukaryotes.

The α-complementation of β-galactosidase was firstly discovered by Jacob and Monod [32]. After that, α-complementation has been proved to function in vivo in bacteria, *S. cerevisiae* and in mammalian cells [33, 43, 44]. In this study, we successfully developed the α-complementation system of β-galactosidase in *P. pastoris*, where only successful mating cells have β-galactosidase activity when α-peptide and ω-peptide are expressed separately in cells of the opposite mating type. This system can be used as a marker for a variety of in vivo studies in *P. pastoris*, such as (i) protein folding and assembly; (ii) protein–protein interactions; (iii) protein trafficking, where only co-compartmentalization shall produce the α-complementation; (iv) processing of cleavage sites by proteases; and (v) easy monitoring of mating in *P. pastoris*. Thus, we believe that this system will become the basis of many experiments and applications in *P. pastoris* systems.

In this study, we used the α-peptide of β-galactosidase with the optimal length (1–92 amino acids) as the second reporter in bicistronic reporter vector to screen functional IRESes in *P. pastoris.* On total, seven IRESes from viruses of TEV, PVY, TRV, RhPV, KSHV crTMV and 5′-UTR of *YAP1* gene were found to internally initiate expression of the second reporter in *P. pastoris* from the 34 selected IRESes. IRESes of TEV and PVY are more active than the other IRESes. In the yeast *S. cerevisiae,* the 5′ UTRs of TFIID, HAP4, *p150* (also called TIF4631) and *YAP1* genes were reported with the ability to initiate internal translation [45–47]. The 5′ UTRs of TFIID, HAP4, p150 and YAP1 were examined for the activity of IRES in *P. pastoris*, and only the 5′ UTR of YAP1 showed the ability to initiate internal translation. Our result indicated that the mechanism of cap-independent translation between *S. cerevisiae* and *P. pastoris* is different. The IRES from EMCV was reported to drive the translation of the second reporter, bacterial hygromycin B phosphotransferase (hph), as internal initiation element in *P. pastoris* [48]. However, we did not detect the activity of EMCV-IRES using our reporter system.

There are two modes of translation initiation: cap-dependent and cap-independent initiation of protein translation. In cap-independent initiation, the presence of the m7G structure at the 5′ terminus of the mRNA is not required. IRES has the ability to directly recruit ribosomes to initiate protein translation by forming special structures. Due to the property, IRESes are often used to express more than one gene in a dicistronic or polycistronic manner under a promoter control from one vector by prokaryotic-like polycistronic expression system. These IRESes screened in this study can be used not only in metabolic engineering and synthetic biology, which often required the expression of entire heterologous pathways, but also as models for studying cap-independent initiation of protein synthesis in *P. pastoris*.

## Conclusions

In this study, we found that *LacZ* gene has background activity in *P. pastoris*. In order to eliminate the background activity of *LacZ* gene, we successfully developed the α-complementation system of β-galactosidase in *P. pastoris*. Co-expression of α- and ω-peptides recovers the enzymatic activity that is absent in the single components. The optimum length of the α-complementing peptide is ~ 92 amino acids. This α-complementation system provides useful tool for future in vivo studies in *P. pastoris* including protein folding and assembly, protein–protein interactions, protein trafficking, processing of cleavage sites by proteases and monitoring cell mating. The α-peptide (1–92aa) was then used as the second reporter in bicistronic vector to screen functional IRESes in *P. pastoris.* A total of seven IRESes from viruses of TEV, PVY, TRV, RhPV, KSHV crTMV and 5′-UTR of *YAP1* gene were found to be functional in *P. pastoris* from the 34 selected IRESes. The two IRESes from TEV and PVY viruses are the most active ones. These functional IRESes can be used to introduce multiple genes into *P. pastoris* by a prokaryotic-like polycistronic manner, which are convenient for researches on metabolic engineering and synthetic biology using *P. pastoris*.

## Materials and methods

### Strains

*Pichia pastoris* GS115 is the host cell for expression, and *Escherichia coli* (*E. coli*) DH5α is the cloning host cell for genetic manipulation.

### Plasmids

To carry out α-complementary of β-galactosidase in *P. pastoris*, a series of vectors were constructed. The *LacZ* gene was firstly amplified from pAd/CMV/V5-GW/LacZ and cloned into pPICZA at EcoRI site for construction of pPICZA-LacZ. N-terminal α-peptide encoding sequences with different lengths (encoding 1 to 33, 1 to 41, 1 to 92, 1 to 142 and 1 to 200 amino acids) and corresponding C-terminal ω-peptide sequences (encoding 34 to 1029, 42 to 1029, 93 to 1029 and 201 to 1029 amino acids) of β-galactosidase were amplified from plasmid pPICZA-LacZ using primers listed in Additional file 2: Table S1 and inserted into expression vectors pAO815 and pPICZA at *Eco*R I using a ClonExpress^®^ II One Step Cloning Kit (Vazyme Biotech; Cat no. C112), respectively.

To screen functional IRES elements in *P. pastoris*, *EGFP* gene and LacZ (1–92) encoding 1 to 92 amino acids of β-galactosidase were used as the first and second cistrons, respectively. pPICZA plasmid was used to construct the dicistronic reporter vector. The *EGFP* gene and LacZ (1–92) were amplified from plasmid pEGFP-N1 and pPICZA-LacZ, respectively. The two fragments were assembled into one fragment using overlapping extension PCR (OE-PCR) method. A SnaBI site was introduced between EGFP and LacZ (1–92), which was used to clone IRES sequences into the dicistronic reporter vector. The assembled fragment EGFP-LacZ (1–92) was cloned into EcoRI site of pAO815 to form plasmid pAO815-EGFP-LacZ (1–92). All IRES elements in this study were synthesized with flanking ACGAGCTGTACAAGTAATAC in 5′ end and ACGACGGGATCTATCATTAC in 3′ end by Sangon Biotech (Shanghai) Co., Ltd., and subcloned into the SnaBI site of pAO815-EGFP-LacZ (1–92) to form the plasmid pAO815-EGFP-IRES-LacZ (1–92) using a ClonExpress^®^ II One Step Cloning Kit.

### Electroporation of *P. pastoris*

The *P. pastoris* GS115 cells were grown on a YPD plate (1% yeast extract, 2% tryptone, 2% dextrose and 1.5% agar) to isolate single colonies. A single colony of GS115 was picked from YPD plate and incubated at 30 °C overnight. 0.1 ml of the overnight culture was incubated in 100 ml fresh medium in a 500-ml flask to OD_600_ ≈ 1.5 before preparing competent cells. The competent cells were prepared with 1 M sorbitol solution according to Pichia Expression Kit Instruction Manual. The purified plasmid DNA was digested with restriction enzyme recommended by instruction manual to obtain linear plasmid DNA. The competent cells were mixed with 10 μg linear plasmid DNA and then add them to a 0.2 cm electroporation cuvette which was incubated about 5 min on the ice and placed in Bio-Rad Gene Pulser (Bio-Rad, USA) to electroporate with 25 vF, 200 Ω. Pre-chilled 1 M sorbitol was added immediately to the electroporation cuvette after pulsing, and the cells were transferred to a sterile 1.5-ml tube. After incubating at 30 °C for 1 h, the transformed cells were spread on MD (2% dextrose, 1.34% yeast nitrogen base, 4 × 10^−5^ % biotin and 1.5% agar) plates. The plates were incubated at 30 °C for 2–3 days until colonies appear.

### Expression of the reporters in *P. pastoris*

Colonies of the successfully transformed *P. pastoris* cells were picked and incubated in a shaking incubator at 220 rpm in BMGY (1% yeast extract, 2% tryptone, 1.34% YNB, 1% glycerol, 100 mM potassium phosphate (pH 6.0) and 4 × 10^−5^% biotin) medium for about 24 h at 30 °C until the OD_600_ reached 2–5. After centrifugation at 3000 rpm for 5 min at room temperature, the supernatant was removed, and the cells were resuspended in BMMY (1% yeast extract, 2% tryptone, 1.34% YNB, 1% methanol, 100 mM potassium phosphate (pH 6.0) and 4 × 10^−5^% biotin) medium to OD_600_ ≈ 1 and incubated under the same conditions for 3 days to induce expression of the reporters. After induction, cells were harvested for further experimental studies.

### Western blot

Harvested cells were washed once with 1-ml 1 M sorbitol, and the cells were resuspended by adding 100 μl breaking buffer (50 mM sodium phosphate, pH 7.4, 1 mM PMSF, 1 mM EDTA and 5% glycerol). An equal volume of acid-washed glass beads (Sigma; Cat no. G8772) was added to the resuspension. The mixture was vortexed for 30 s and then immediately placed on ice for 30 s. This operation was repeated eight times. The cell lysate was transferred to a new tube after centrifuging at 16,000 rpm for 10 min at 4 °C. Protein concentration was quantified by using Pierce™ BCA Protein Assay Kit (Thermo; Cat no. 23227) and then boiled and denatured at 95 °C for 5 min. For western blot analysis, the 30 μg samples were separated on 10% SDS-PAGE, and protein bands were transferred to Hybond-C nitrocellulose membrane (Amersham Bioscience, Little Chalfont, UK) through electroblotting. The membranes were blocked with 5% fat-free milk and probed overnight at 4 °C with primary antibody, against EGFP (Proteintech, China, Cat no. 50430-2-AP), and IRDye 800CW-conjugated goat anti-rabbit secondary antibodies (LI–COR Biosciences, Lincoln, NE, USA; cat. no. C60607–15) were used as the secondary antibody at 1:1000 dilutions. The signals were detected and measured using LICOR Odyssey system (LI–COR, Nebraska, USA).

### Assay for β-galactosidase activity

For qualitative analysis of β-galactosidase activity, BMMY plates containing 200 μg/ml of X-Gal were prepared. Yeast transformants were transferred into BMMY plates with X-Gal from YPD plates using sterile toothpicks, and BMMY plates were grown for about 3 days at 30 °C until the blue colonies appeared.

For quantitative analysis of β-galactosidase activity, single colonies of transformants from YPD plates were grown at 30 °C for 24 h in 5 ml of BMGY. Cells were harvested and transferred into 5 ml of BMMY to grow at 30 °C until an optical density at 600 nm of about 1.0 was reached. Cells were harvested by centrifugation and washed using 1.0-ml sterile dH_2_O. After washing, cells were pelleted again and resuspended in breaking buffer (100 mM Tris–HCl, 1 mM dithiothreitol, 20% glycerol, pH 8.0). Glass beads were added into the tube with the cells in breaking buffer. Cell lysis was performed by vortexing six times at top speed in 15-s bursts (chilling on ice between bursts). The extract was clarified by centrifugation, and total protein concentration was determined by Bradford assay Pierce™ BCA Protein Assay Kit. Forty micrograms of total proteins was added to 500 μl Z buffer. In total, 200 μl of ONPG (4 mg/ml) was added to initiate the reaction at 28 °C. The reaction was stopped by adding Na_2_CO_3_ solution. Absorbance was determined at 420 nm. The results were normalized against protein concentration and incubation time.

### RT-PCR

Total RNA was isolated from *P. pastoris* cells using the Yeast RNA Kit (Omega; cat. no. R6870). The cDNA was obtained by reverse transcription of total RNA according to the instructions of the GoScript™ Reverse Transcription Mix, Oligo (dT) kit (Promega; cat. no. A2790). The RT-PCR reaction system was prepared using a 2× M5 HiPer Taq PCR mix (Mei5bio; cat. no. MF001-01), and RT-PCR was performed in a SimpliAmp Thermal Cycler (Applied biosystems, USA). The primers used in RT-PCR are shown in Additional file 2: Table S1.

## Supplementary information

**Additional file 1.** Sequences of IRES.

**Additional file 2.** Additional Table and Figure.

## Data Availability

The data supporting the conclusions of this article are included with the article and its additional file.

## Abbreviations

IRES: internal ribosome entry site
UTR: untranslated region
EGFP: enhanced green fluorescent protein
X-gal: 5-bromo-4-chloro-3-indolyl β-d-galactoside
OE-PCR: overlapping extension PCR
ONPG: 2-nitrophenyl β-d-galactopyranoside
TEV: Tobacco etch virus
PVY: Potato virus Y
RhPV: Rhopalosiphum padi
TRV: Triatoma virus
KSHV: Kaposi-sarcoma-associated herpesvirus
crTMV: Tobacco mosaic virus
