# Supplementary material for: Screening for functional IRESes using α-complementation system of β-galactosidase in Pichia pastoris
Source: Biotechnol Biofuels. 2019 Dec 27;12:300. doi: 10.1186/s13068-019-1640-3 (PMC6933714; doi:10.1186/s13068-019-1640-3)
Supplement: Supplementary file 1 — Additional file 1. Sequences of IRES. [file 13068_2019_1640_MOESM1_ESM.docx]

Sequences of IRES:

1. bvdv (385bp)

GTATACGAGGTTAGGCAAGTTCTCGTATACATATTGGACACTCTAAAAATAATTAGGCCTAGGGGACAAAAATCCTCCTTAGCGAAGGCCGAAAAGAGGCTAACCATGCCCTTAGTAGGACTAGCAAAATAAGGGGGGTAGCAACAGTGGCGAGTTCGTTGGATGGCTGAAGCCCTGAGTACAGGGTAGTCGTCAGTGGTTCGACGCTTTGGAGGACAAGCCTCGAGATGCCACGTGGACGAGGGCATGCCCACAGCACATCTTAACCTGGACAGGGGTCGTTCAGGTGAAAACGGTTTAACCAACCGCTACGAATACAGTCTGATAGGATGCTGCAGAGGCCCACTGTATTGCTACTGAAAATCTCTGCTGTACATGGCACATG

1. cpmv (354bp)

ATGTTTTCTTTCACTGAAGCGAAATCAAAGATCTCTTTGTGGACACGTAGTGCGGCGCCATTAAATAACGTGTACTTGTCCTATTCTTGTCGGTGTGGTCTTGGGAAAAGAAAGCTTGCTGGAGGCTGCTGTTCAGCCCCATACATTACTTGTTACGATTCTGCTGACTTTCGGCGGGTGCAATATCTCTACTTCTGCTTGACGAGGTATTGTTGCCTGTACTTCTTTCTTCTTCTTCTTGCTGATTGGTTCTATAAGAAATCTAGTATTTTCTTTGAAACAGAGTTTTCCCGTGGTTTTCGAACTTGGAGAAAGATTGTTAAGCTTCTGTATATTCTGCCCAAATTTGAAATG

1. CrPV (195bp)

AAAGCAAAAATGTGATCTTGCTTGTAAATACAATTTTGAGAGGTTAATAAATTACAAGTAGTGCTATTTTTGTATTTAGGTTAGCTATTTAGCTTTACGTTCCAGGATGCCTAGTGGCAGCCCCACAATATCCAGGAAGCCCTCTCTGCGGTTTTTCAGATTAGGTAGTCGAAAAACCTAAGAAATTTACCTgct

1. CrTMV (151bp)

GAATTCGTCGATTCGGTTGCAGCATTTAAAGCGGTTGACAACTTTAAAAGAAGGAAAAAGAAGGTTGAAGAAAAGGGTGTAGTAAGTAAGTATAAGTACAGACCGGAGAAGTACGCCGGTCCTGATTCGTTTAATTTGAAAGAAGAAAATG

1. csfv (1-376)

GTATACGAGGTTAGTTCATTCTCGTATGCATGATTGGACAAATCAAAATTTCAATTTGGTTCAGGGCCTCCCTCCAGCGACGGCCGAACTGGGCTAGCCATGCCCACAGTAGGACTAGCAAACGGAGGGACTAGCCGTAGTGGCGAGCTCCCTGGGTGGTCTAAGTCCTGAGTACAGGACAGTCGTCAGTAGTTCGACGTGAGCAGAAGCCCACCTCGAGATGCTATGTGGACGAGGGCATGCCCAAGACACACCTTAACCCTAGCGGGGGTCGCTAGGGTGAAATCACACCACGTGATGGGAGTACGACCTGATAGGGCGCTGCAGAGGCCCACTATTAGGCTAGTATAAAAATCTCTGCTGTACATGGCACATG

1. DCV (187bp)

TAAGATGTGATCTTGCTTCCTTATACAATTTTGAGAGGTTAATAAGAAGGAAGTAGTGCTATCTTAATAATTAGGTTAACTATTTAGTTTTACTGTTCAGGATGCCTATTGGCAGCCCCATAATATCCAGGACACCCTCTCTGCTTCTTATATGATTAGGTTGTCATTTAGAATAAGAAAATAACCT

1. EMCV (577bp)

CCCCTCTCCCTCCCCCCCCCCTAACGTTACTGGCCGAAGCCGCTTGGAATAAGGCCGGTGTGCGTTTGTCTATATGTTATTTTCCACCATATTGCCGTCTTTTGGCAATGTGAGGGCCCGGAAACCTGGCCCTGTCTTCTTGACGAGCATTCCTAGGGGTCTTTCCCCTCTCGCCAAAGGAATGCAAGGTCTGTTGAATGTCGTGAAGGAAGCAGTTCCTCTGGAAGCTTCTTGAAGACAAACAACGTCTGTAGCGACCCTTTGCAGGCAGCGGAACCCCCCACCTGGCGACAGGTGCCTCTGCGGCCAAAAGCCACGTGTATAAGATACACCTGCAAAGGCGGCACAACCCCAGTGCCACGTTGTGAGTTGGATAGTTGTGGAAAGAGTCAAATGGCTCTCCTCAAGCGTATTCAACAAGGGGCTGAAGGATGCCCAGAAGGTACCCCATTGTATGGGATCTGATCTGGGGCCTCGGTGCACATGCTTTACATGTGTTTAGTCGAGGTTAAAAAACGTCTAGGCCCCCCGAACCACGGGGACGTGGTTTTCCTTTGAAAAACACGATGATAATATG

1. FMDV (465bp)

ATGCAGGTTTCCCCAACTGACACAAACCGTGCAACTTGAAACTCCGCCTGGTCTTTCCAGGTCTAGAGGGGTAACATTTTGTACTGTGTTTGACTCCACGCTCGATCCACTAGCGAGTGTTAGTAGCGGTACTGCTGTCTCGTAGCGGAGCATGTTGGCCGTGGGAACACCTCCTTGGTAACAAGGACCCACGGGGCCGAAAGCCATGTCCTAACGGACCCAACATGTGTGCAACCCCAGCACGGCAGCTTTACTGTGAAACCCACTTCAAGGTGACATTGATACTGGTACTCAAACACTGGTGACAGGCTAAGGATGCCCTTCAGGTACCCCGAGGTAACAAGCGACACTCGGGATCTGAGAAGGGGACTGGGACTTCTTTAAAGTGCCCAGTTTAAAAAGCTTCTACGCCTGAATAGGTGACCGGAGGCCGGCACCTTTCCTTTTATAACCACTGAACACATG

1. F-Mulv-g (357bp)

GCGCCAGTCCTCCGATAGACTGAGTCGCCCGGGTACCCGTATTCCCAATAAAGCCTCTTGCTGTTGCATCCGACTCGTGGTCTCGCTGTTCCTTGGGAGGGTCTCCTCAGAGTGATTGACTACCCGTCTCGGGGGTCTTTCATTTGGGGGCTCGTCCGGGATCTGGAGACCCTTGCCCAAGGACCACCGACCCACCACCGGGAGGTAAGCTGGCCAGCAATTGATCTGTGTCTGTCCATTGTCCCGTGTCTTTGATTGATTTTATGCGCCTGCGTTTGTACTAGTTGGCCGACTAGCTCTGTACCTGGCGGACCCGTGGTGGAACTGACGGGTTCGAGACACCCGGCCGCAACCCTG

1. F-MuLV-e (289bp)

CTCATTTACAGGCACTCTACCTGGTCCAGCACGAAGTCTGGAGACCGTTGGCGGCAGCTTACCAAGAGCAACTAGACCGGCCGGTAGTACCTCACCCTTTCCGGGTCGGTGACACAGTGTGGGTCCGCAGACACCAAACTAAAAATCTAGAACCCCGCTGGAAAGGACCCTATACCGTCCTACTGACTACCCCCACCGCTCTCAAAGTAGACGGCATTGCAGCGTGGATCCACGCTGCCCACGTAAAGGCTGCCGACACCAGGATTGAGCCACCAGCAGAATCGACATG

1. GBV-B (426bp)

ACACTCCGCTAGGAATGCTCCTGGAGCACCCCCCCTAGCAGGGCGTGGGGGATTTCCCCTGCCCGTCTGCAGAAGGGTGGAGCCAACCACCTTAGTATGTAGGCGGCGGGACTCATGACGCTCGCGTGATGACAAGCGCCAAGCTTGACTTGGATGGCCCTGATGGGCGTTCATGGGTTCGGTGGTGGTGGCGCTTTAGGCAGCCTCCACGCCCACCACCTCCCAGATAGAGCGGCGGCACTGTAGGGAAGACCGGGGACCGGTCACTACCAAGGACGCAGACCTCTTTTTGAGTATCACGCCTCCGGAAGTAGTTGGGCAAGCCCACCTATATGTGTTGGGATGGTTGGGGTTAGCCATCCATACCGTACTGCCTGATAGGGTCCTTGCGAGGGGATCTGGGAGTCTCGTAGACCGTAGCACATG

1. GLV (1-369bp)

GGAAGGAGTGCCAGGCCATTACCTTCCGCCCCTGCCTAACAAAACTGGCTCGCCAGAGGCGGGGGGGCGTGGTAGACGGTATGTCTAGGTCCAGAGAAAGCGGCCGCTGTTAGGCTGCTAGACTTCGCCACGCTTACACCATTGAGAACACATAGGTGGTGTAATATTATGTGTTTGGCTGCGTGTGTCACGAGCACGATCTATGGGGGAATCTAACCCAGGTGGTGTTGTGTATGATGAGGTACAAGCCCGTCAACACCACAGGTGGAGAGGGACAGGAGTCTCATTTCCCCTCCAAAAAACAGCAGTGGTCCGCCCCTCATGCCCTATGAAACATTAGGGGGCCGACACTAGTGGGCAAGCCCGATG

hamsvvl30-IRES GI:(207672 (25-543))

1. HaMSV (529bp)

CGATCGATCAATAGGCTCAGATCTGGGGACTATCTGGGCGGGCCAGAGAAGGAGCTGACGAGCTCGGACTTCTCCCCCGCAGCCCTAGAAGACGTTCCAAGGGTGGTTGGAGGAGAGGGAGATCCGGATCCGTGGCACCTCCGTCCGTTTTCGGAGGGATCCGCACCCTTGATGACTCCGTCTGAATTTTTTGGTTTCAGTTTGGTACCGAAGCTGCGCGGCTCTGCTTGTTACTTGTTTGACTGTTGGAATTGTTTGTCTTCTTTGTGACCTGACTGTGGTTTTCTGGACGTGTTGTGTCTGTTAGTGTCTTTTTGACTTTTGTTTCGTGTTTGAATTTGGACTGACGACTGTGTTTAAAATCTTGGACCGACGACTGTGTTTGAAATCATGAAACTGTTTGCTTTGTTCGTCGAAGAGTTTTACTTGGTCCCCTTAACGCTTAGTGAGTAAGAAACTTAATTTTGTAGAGACCCCGCTCTAGTGGCAGTGTGTTGGTTGATAGCCAAAGTTAATTTTTAAAACATAG

1. HAV (571bp)

TCAGGGTTCTTAAATCTGTTTCTCTATAAGAACACTCATTTCACGCTTTCTGTCTTCTTTCTTCCAGGGCTCTCCCCTTGCCCTAGGCTCTGGCCGTTGCGCCCGGCGGGGTCAACTCCATGATTAGCATGGAGCTGTAGGAGTCTAAATTGGGGACACAGATGTTTGGAACGTCACCTTGCAGTGTTAACTTGGCTTTCATGAATCTCTTTGATCTTCCACAAGGGGTAGGCTACGGGTGAAACCTCTTAGGCTAATACTTCTATGAAGAGATGCCTTGGATAGGGTAACAGCGGCGGATATTGGTGAGTTGTTAAGACAAAAACCATTCAACGCCGGAGGACTGACTCTCATCCAGTGGATGCATTGAGTGGATTGACTGTCGGGGCTGTCTTTAGGCTTAATTCCAGACCTCTCTGTGCTTGGGGCAAACATCATTTGGCCTTAAATGGGATTCTGTGAGAGGGGATCCCTCCATTGCCAGCTGGACTGTTCTTTGGGGCCTTATGTGGTGTTTGCCGCTGAGGTACTCAGGGGCATTTAGGTTTTTCCTCATTCTTAAATAATAATG

1. HCV (344bp)

GCCAGCCCCCGATTGGGGGCGACACTCCACCATAGATCACTCCCCTGTGAGGAACTACTGTCTTCACGCAGAAAGCGTCTAGCCATGGCGTTAGTATGAGTGTCGTGCAGCCTCCAGGACCCCCCCTCCCGGGAGAGCCATAGTGGTCTGCGGAACCGGTGAGTACACCGGAATTGCCAGGACGACCGGGTCCTTTCTTGGATCAACCCGCTCAATGCCTGGAGATTTGGGCGTGCCCCCGCGAGACTGCTAGCCGAGTAGTGTTGGGTCGCGAAAGGCCTTGTGGTACTGCCTGATAGGGTGCTTGCGAGTGCCCCGGGAGGTCTCGTAGACCGTGCATCATG

1. HIV1 (474bp)

GATGGGTGCGAGAGCGTCAGTATTAAGCGGGGGAGAATTAGATCGATGGGAAAAAATTCGGTTAAGGCCAGGGGGAAAGAAAAAATATAAATTAAAACATATAGTATGGGCAAGCAGGGAGCTAGAACGATTCGCAGTTAATCCTGGCCTGTTAGAAACATCAGAAGGCTGTAGACAAATACTGGGACAGCTACAACCATCCCTTCAGACAGGATCAGAAGAACTTAGATCATTATATAATACAGTAGCAACCCTCTATTGTGTGCATCAAAGGATAGAGATAAAAGACACCAAGGAAGCTTTAGACAAGATAGAGGAAGAGCAAAACAAAAGTAAGAAAAAAGCACAGCAAGCAGCAGCTGACACAGGACACAGCAATCAGGTCAGCCAAAATTACCCTATAGTGCAGAACATCCAGGGGCAAATGGTACATCAGGCCATATCACCTAGAACTTTAAATGCATGGGTAAAAGT

1. htlv-1 (268bp)

GGCTCGCATCTCTCCTTCACGCGCCCGCCGCCTTACCTGAGGCCGCCATCCACGCCGGTTGAGTCGCGTTCTGCCGCCTCCCGCCTGTGGTGCCTCCTGAACTACGTCCGCCGTCTAGGTAAGTTTAGAGCTCAGGTCGAGACCGGGCCTTTGTCCGGCGCTCCCTTGGAGCCTACCTAGACTCAGCCGGCTCTCCACGCTTTGCCTGACCCTGCTTGCTCAACTCTACGTCTTTGTTTCGTTTTCTGTTCTGCGCCGTTACAGATCG

1. kshv (493bp)

CGGCAGACTCCTTTTCCCGCCAAGAACTTATAGACCAGGAGAAAGAACTCCTTGAGAAGTTGGCGTGGCGAACAGAGGCAGTCTTAGCGACGGACGTCACTTCCTTCTTGTTACTTAAATTGCTGGGGGGCTCCCAACACCTGGACTTTTGGCACCACGAGGTCAACACCCTGATTACAAAAGCCTTAGTTGACCCAAAGACTGGCTCATTGCCCGCCTCTATTATCAGCGCTGCAGGCTGTGCGCTGTTGGTTCCTGCCAACGTCATTCCGCAGGATACCCACTCGGGTGGGGTAGTTCCTCAGCTGGCAGCATATTGGGATGCGATGTTTCCGTTCTACAGGCGGCAGTGGAACAGATCCTAACATCTGTTTCGGACTTTGATCTGCGCATTCTGGACAGCTATTAAGCTTGTGATTTTGTTTAGGGCGGAAAAATAATTTTCCTTTGTTTTTCCACATCGGTGCCTTCACATATACAAGCCGGCACCATG

1. MoMuLV (129bp)

TAAAACAGTTCCCGCCTCCGTCTGAATTTTTGCTTTCGGTTTGGAACCGAAGCCGCGCGTCTTGTCTGCTGCAGCATCGTTCTGTGTTGTCTCTGTCTGACTGTGTTTCTGTATTTGTCTGAAAATATG

1. PLRV (216bp)

ACTGGGCTGATGATTATGACTCCGATGAGGATTACGGTCTGGAGAGAGAGGCTGCAACAAATGCGCCCGCAGAGAAAACTGCTCAAACAAACTCAGCAGAGAAGACTGCTCCATCAACTTCAGCAGAGAAAACTGCTCCAACAAACAAGCCTTTAAATGGGCAAGCGGCACCGTCCGCCAAAACAAACGGCAACTCCGACATCCCCGACGCCGCTA

1. PSIV (247bp)

TCGACACGCGGCCTTCCAAGCAGTTAGGGAAACCGACTTCTTTGAAGAAGAAAGCTGACTATGTGATCTTATTAAAATTAGGTTAAATTTCGAGGTTAAAAATAGTTTTAATATTGCTATAGTCTTAGAGGTCTTGTATATTTATACTTACCACACAAGATGGACCGGAGCAGCCCTCCAATATCTAGTGTACCCTCGTGCTCGCTCAAACATTAAGTGGTGTTGTGCGAAAAGAATCTCACTTATG

1. PTV-1 (289bp)

ACTTGGTTATGAATTCATTGGATTAACCCCTCTGAAAGACCTGCTCTGGCGCGAGCTAAAGCGCAATTGTCACCAGGTATTGCACCAATGGTGGCGACAGGGTACAGAAGAGCAAGTACTCCTGACTGGGTAATGGGACTGCATTGCATATCCCTAGGCACCTATTGAGATTTCTCTGGGGCCCACCAGCGTGGAGTTCCTGTATGGGAATGCAGGACTGGACTTGTGCTGCCTGACAGGGTCGCGGCTGGCCGTCTGTACTTTGTATAGTCAGTTGAAACTCACCATG

1. PVY (187bp)

AATTAAAACAACTCAATACAACATAAGAAAAACAACGCAAAAACACTCATAAACGCTCATTCTCACTCAAGCAACTTGCTAAGTTTCAGTTTAAATCATTTCCTTGCAATTCTCTAGAACAATATTGGAAACCATTTCAACTCAACAAGCAATTTCATCACTTCCAACCAATTTCAGATCCTCAATG

1. RhPV (220bp)

AATAACATATATATGTCAACCCTGCTCATTGGTTTAATTGAGCGCATTTAGTGTTGTGTGATCTTGCGCGATAAATGCTGACGTGAAAACGTTGCGTATTGCTACAACACTTGGTTAGCTATTTAGCTTTACTAATCAAGACGCCGTCGTGCAGCCCACAAAAGTCTAGATACGTCACAGGAGAGCATACGCTAGGTCGCGTTGACTATCCTTATATATG

1. RSV (153bp)

GGCAGAAGCTGAGTGGCGTCGGAGGGAGCTCTACTGCAGGGAGCCCAGATACCCTACCGAGAACTCAGAGAGTCGTTGGAAGACGGGAAGGAAGCCCGACGACTGAGCAGTCCACCCCAGGCGTGATTCTGGTCGCCCGGTGGATCAAGCATG

1. SIV (477bp)

GTAGAGCCTGGGTGTTCCCTGCTAGACTCTCACCAGCACTTGGCCAGTGCTGGGCAGAGTGGCTCCACGCTTGCTTGCTTAAAGACCTCTTCAATAAAGCTGCCATTTTAGAAGTAAGCCAGTGTGTGTTCCCATCTCTCCTAGTCGCCGCCTGGTCAACTCGGTACTCGGTAATAAGAAGACCCTGGTCTGTTAGGACCCTTTCTGCTTTGAGAAACCGAAGCAGGAAAATCCCTAGCAGATTGGCGCCCGAACAGGACTTGAAGGAGAGTGAGAGACTCCTGAGTACGGCTGAGTGAAGGCAGTAAGGGCGGCAGGAACCAACCACGACGGAGTGCTCCTATAAAGGCGCGGGTCGGTACCAGACGGCGTGAGGAGCGGGAGAGGAGGAGGCCTCCGGTTGCAGGTAAGTGCAACACAAAAAAGAAATAGCTGTCTTGTTATCCAGGAAGGGATAATAAGATAGAGTGGGAGATG

1. TEV (146bp)

AAATAACAAATCTCAACACAACATATACAAAACAAACGAATCTCAAGCAATCAAGCATTCTACTTCTATTGCAGCAATTTAAATCATTTCTTTTAAAGCAAAAGCAATTTTCTGAAAATTTTCACCATTTACGAACGATAGCAATG

1. TRV (551bp)

CAAAATTGCGTGCGAGAAAGCACGCAAATCAAAGTCTAGTGCGTAATTCACTCACTACCGGCGTAATTTGTGGTTATGCTATTGCGTTGAGAGTGTTGTGGGCGTAGTACGGGGGCTTTTGGTTTGTGTGTGATTAATATGCATTCCCAGTTTTGTTTAGTTTTAGATTACTGATTTATTTTTCGAACTACCCGAATTTATTTAGGCTCTTCGAGAAATAATGATGAACTGTCCTCAACAAGTATGAAAAGCAATTATTTGTGAAGTTACTTGTTGTTGTAAGATTATTGACCTCTTAGATTTTTCTAAGTTGTAATGCTTTGTTTTCTGATTGACTAGATTATGAATCCAATTAAAAGGAGTAGTGGTCTAATATAGTCTGTGTGACCTGCAGGCATTTTGTGAAAAGGGTAAAGTATGAAAGCTACTCTCAGAAAAGTACTTATGTATTGATGAGAGCCTTAAAATGACTTTATATTCACAAAACTGCTGGAAGACAATGATCTGGGGTATCACATTCCCTCTTAGGTTAAGTTTCGCACTACTAGGAA

1. trv-igr (178bp)

TGATCTTGCTTTCGTAATAAAATTCTGTACATAAAAGTCGAAAGTATTGCTATAGTTAAGGTTGCGCTTGCCTATTTAGGCATACTTCTCAGGATGGCGCGTTGCAGTCCAACAAGATCCAGGGACTGTACAGAATTTTCCTATACCTCGAGTCGGGTTTGGAATCTAAGGTTGACTC

1. TSV (212bp)

TAGCACCACCCGATCGTAAACTCCATGTATTGGTTACCCATCTGCATCGAAAACTCTCCGAACACTAGGTGCAGTAAGGCTTTCATGGAGTGGTTTGCTATTTAGCGTACGTGTACCATAGGCAGCCCCAAAAACACGTGTGAGGAGAAAGTCCCAGTCACTTTGGGCAAAGTAGACAGCCGCGCTTGCGTGGTGGGACTTAATTAATGCCT

1. YAP1 (373bp)

GAATTCGGATAGTAACCAGCCCTAGCTGTTTGGTTGATTTGACCTAGGTTACTCTTTTCTTTTTCTGGGTGCGGGTAACAATTTGGGCCCCGCAAAGCGCCGTCTTTGTCATGGGAACCGGAAACCCTCCGATGAAGAGTAGGAGGGTGGCAACTGATGGATGCGTAAGGTCTTAAGAGATACATTTGATTAATAGTCTTCCGTTTACCGATTAAGCACAGTACCTTTACGTTATATATAGGATTGGTGTTTAGCTTTTTTTCCTGAGCCCCTGGTTGACTTGTGCATGAACACGAGCCATTTTTAGTTTGTTTAAGGGAAGTTTTTTGCCACCCAAAACGTTTAAAGAAGAAAAGTTGTTTCTTAAACCATG

1. P150 (531bp)

CCCAGTTCGATCCTGGGCGAAATCATTTTTTTGAAAATTACATTAATAAGGCTTTTTTCAATATCTCTGGAACAACAGTTTGTTTCTACTTACTAATAGCTTTAAGGACCCTCTTGGACATCATGATGGCAGACTTCCATCGTAGTGGGATGATCATATGATGGGCGCTATCCTCATCGCGACTCGATAACGACGTGAGAAACGATTTTTTTTTTTCTTTTTCACCGTATTTTTGTGCGTCCTTTTTCAATTATAGCTTTTTTTTATTTTTTTTTTTTCTCGTACTGTTTCACTGACAAAAGTTTTTTTTCAAGAAAAATTTTCGATGCCGCGTTCTCTGTGTGCAACGGATGGATGGTAGATGGAATTTCAATATGTTGCTTGAAATTTTACCAATCTTGATATTGTGATAATTTACTTAATTATGATTCTTCCTCTTCCCTTCAATTTCTTAAAGCTTCTTACTTTACTCCTTCTTGCTCATAAATAAGCAAGGTAAGAGGACAACTGTAATTACCTATTACAATAATG

1. HAP4 (503bp)

ATCGATTTTGCAGATTGTTCTAAAAGTAAATGGATTGCTATTTTCTTTCCGAGACTACTCTAAAAAAATTTATTGAGTATGAGATCGTTTTTAGATAAATTATATATATTGTAAAGCTATTAACTAATCTCCTATATCAATTTCTTCTTGCTTAACCCCGTGTGGTTGTTTAGGTCCATCTCCTTTTTCCTTTTAATTTTTTTACCTTTATTAATTCCTTCACCTCTCTAAACCCCAGTTTTATATCGTATATGCTATCTACAGGTCCACTTTACACTTAATAATATAAAAATACTACTATAAAGGAACCAGAAAAATAAAAAAGGGTCATTATTTATTTGAGCAGATCATTATCAAACGCATAGGAAGAGAAAAAACACAGTTTTATTTTTTTTCCACACATATTTATTGGTCTCCTAGTACATCAAAGAGCATTTTAATGGGTTGCTGATTTGTTTTACCTACATTTTCTAGTACAAAAAAAAAACAAAAAAAGAATCATG

1. TFIID (275bp)

AGATCTACATATAAAACATGGCTTCAAAGGATTACTAATGACTTTTTTTACCTTGATAGGTATTCTTGATGGTAAGAGCAAACAAGGGACGTGAAAATTACAGTAGTTACTGTTTTTTTTGGACTATAAGATCGGGGGAAAGATAACACATAAGAAATAAAACGACTACTAGTTAGACTGCTCTGCGGAAGAAGCAAGGAAGTAAAGGCTGCATTTTATTTTTCTTTTCTAGTCCAACATAAACAGGTGTATCAAGAGAAACTTTTTTAATTATG
