# Supplementary material for: Screening for functional IRESes using α-complementation system of β-galactosidase in Pichia pastoris
Source: Biotechnol Biofuels. 2019 Dec 27;12:300. doi: 10.1186/s13068-019-1640-3 (PMC6933714; doi:10.1186/s13068-019-1640-3)
Supplement: Supplementary file 2 — Additional file 2. Additional Table and Figure. [file 13068_2019_1640_MOESM2_ESM.docx]

**Table S1. Primers using in this study**

| **Description** | **Primer sequence (5’–3’)** | **Restriction site** |
| --- | --- | --- |
| **Preparation for α-complementation system** | | |
| Upstream primer for amplifying all α-peptides | GACTGGTTCCAATTGACAAGC |  |
| Downstream primer for amplifying α-peptide (1-33) | CCGCTCGAGttaacgccagctggcgaaagg |  |
| Downstream primer for amplifying α-peptide (1-41) | CCGCTCGAGttaatcggtgcgggcctcttc |  |
| Downstream primer for amplifying α-peptide (1-92) | CCGCTCGAGttaatcgtaaccgtgcatctg |  |
| Downstream primer for amplifying α-peptide (1-200) | CCGCTCGAGttaccgccacatatcctgatcttcc |  |
| Upstream primer for amplifying ω-peptide (34-1029) | CCGGAATTCACCatgaatagcgaagaggcccgcac | *Eco*R I |
| Upstream primer for amplifying ω-peptide (42-1029) | CCGGAATTCACCatgcgcccttcccaacagttgcg | *Eco*R I |
| Upstream primer for amplifying ω-peptide(93-1029) | CCGGAATTCACCatggcgcccatctacaccaacgt | *Eco*R I |
| Upstream primer for amplifying ω-peptide (201-1029) | CCGGAATTCACCatgatgagcggcattttccgtg | *Eco*R I |
| Downstream primer for amplifying all ω-peptide | CCGCTCGAGttatttttgacaccagaccaactgg |  |
| **Preparation for plasmid pAO815-EGFP-IRES-LacZ(1-92)** | | |
| Upstream primer | ACGAGCTGTACAAGTAATAC |  |
| Downstream primer | ACGACGGGATCTATCATTAC |  |
| **Preparation for plasmid pAO815-EGFP-LacZ(1-92) by Overlap extension PCR** | | |
| Upstream primer for amplifying LacZ(1-92) fragment | acaagtaatacgtaatgatagatcccgtcgttttaca | *Sna*B I |
| Downstream primer for amplifying LacZ(1-92) fragment | gtcatgtctaaggcgaattcttaaacgccatcaaaaataa | *Eco*R I |
| Upstream primer for amplifying EGFP fragment | attattcgaaacgaggaattcaccatggtgagcaagggcg | *Eco*R I |
| Downstream primer for amplifying EGFP fragment | tctatcattacgtattacttgtacagctcgtccatgc | *Sna*B I |
| **RT-PCR for exclusion of cryptic splicing** | | |
| Gup | ACCATGGTGAGCAAGGGCGA |  |
| Gdown | CTTGTACAGCTCGTCCATGC |  |
| Iup | gcatggacgagctgtacaag |  |
| Idown | AAAACGACGGGATCTATCAT |  |
| Lup | ATGATAGATCCCGTCGTTTT |  |
| Ldown | CCGCTCGAGTTAATCGTAACCGTGCATCTG |  |


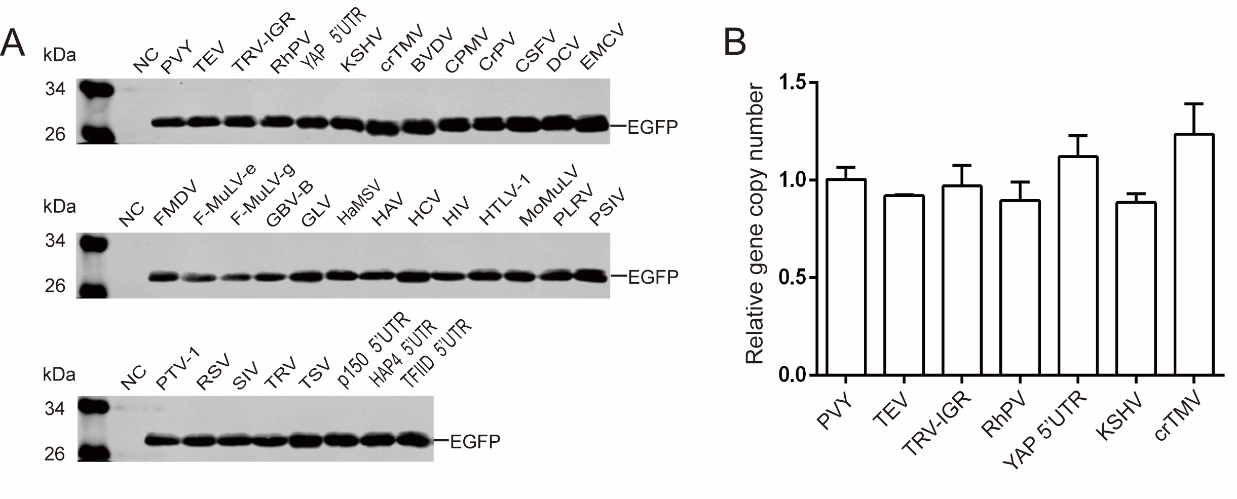


Figure S1. Expression of the first reporter EGFP in 34 IRES vectors (A) and relative copy number of encoding α-peptide (1-92) sequence of 7 functional IRESes in transgenic strains (B).
